# Formulation and Characterization of Nutrient-Dense Medjool Date Bars: Integration of Proteins, Bioactives, and Processing Stability for Functional Snack Innovation

**DOI:** 10.3390/foods15050887

**Published:** 2026-03-05

**Authors:** Ahmed H. Bahloul, Mahmoud H. Mahmoud, Galal A. Ghazal, Hassan Barakat

**Affiliations:** 1 Food Technology Department, Faculty of Agriculture, Benha University, Moshtohor 13736, Egypt; ahmed.bahloul@fagr.bu.edu.eg (A.H.B.); mahmoud.mahmoud@fagr.bu.edu.eg (M.H.M.); galal.ibrahim@fagr.bu.edu.eg (G.A.G.); 2 Department of Food Science and Human Nutrition, College of Agriculture and Food, Qassim University, Buraydah 51452, Saudi Arabia

**Keywords:** Medjool date, energy bars, nutritional quality, phytochemical characterization, texture analysis, food supply

## Abstract

This investigation focused on developing nutrient-dense Medjool date-based bars (MDBs) formulated with Medjool date paste, milk protein concentrate, whey proteins, and other functional ingredients. Comprehensive proximate analysis, mineral profiling, amino acid determination, and instrumental assessments, including color measurement, scanning electron microscopy, differential scanning calorimetry, and texture analysis, were performed, followed by organoleptic evaluation. Medjool date paste served as the primary carbohydrate source (76.44%), while whey protein isolate and milk protein concentrate contributed substantially to the protein fraction (89.26% and 81.62%, respectively). The resulting bars contained 19.32–26.78% crude protein, 10.96% fat, and 12.35–12.71% moisture, delivering 414.72–416.04 Kcal 100 g^−1^. Sugar profiles remained consistent across formulations (total sugars: 36.77–36.98%), with appreciable mineral content including potassium (884–923 mg 100 g^−1^), calcium (418–585 mg 100 g^−1^), and phosphorus (402–459 mg 100 g^−1^). The essential amino acid composition equaled or surpassed that of hen’s egg, establishing the product as a superior protein source. Antioxidant analysis demonstrated total phenolic content of 452.22–554.12 mg GAE 100 g^−1^ and total flavonoids of 358.06–374.24 mg QE 100 g^−1^, with consistent radical scavenging capacity, reduced browning via protein–polyphenol binding (ΔG −58 to −72 kJ mol^−1^), a balanced texture (hardness 157–189 N), and consistent sensory scores (87.63–93.28% acceptability), without significant differences among formulations. Molecular docking confirmed β-lactoglobulin’s tight antioxidant shielding and caseinate’s flexible bioavailability boost, yielding shelf-stable functional snacks that advance date palm valorization. The results demonstrate the successful development of functional MDBs with an excellent nutritional profile and strong panelist acceptance.

## 1. Introduction

Shifts in dietary patterns and rising consumer interest in health-oriented products enriched with functional ingredients have intensified the focus on enhancing the nutritional profiles of many established foods. For a large segment of today’s consumers, being considered healthy or functional has become an essential food purchasing criterion rather than an added benefit. Escalating healthcare costs, longer life expectancy, population aging, and the aspiration to maintain high quality of life have all contributed to the greater demand for foods that satisfy these elevated expectations. Thus, appealing flavor and desirable sensory attributes alone are no longer adequate; food is also expected to deliver meaningful nutritional value. In response, numerous recent investigations have focused on developing new categories of food products with targeted textural, sensory, or functional characteristics [1].

The date palm tree (*Phoenix dactylifera* L., family Arecaceae) has long been recognized as an essential contributor to the regional economy and food supply in the Middle East and North Africa [2]. Egypt is the primary date producer, with 18% of the world production, while Saudi Arabia supplies almost 16% of the world’s date fruit demand [3]. Dates are used in numerous food products, such as energy bars, which have gained popularity [3,4]. Nut flour, almond flour, and almond milk powder have also been used to manufacture date bars [5,6]. Recently, cheddar cheese and whey protein isolate were applied with roasted chickpea flour, rice flour, and dried apricots [7,8]. Most recently, Barakat and Almutairi formulated date-based bars using date paste, cow’s ghee, peanut butter, sesame seeds, ground walnuts, roasted cashews, whole oat powder, oat fiber, skimmed milk powder, and Khalas date syrup [9].

The selection of Medjool as the primary formulation ingredient was driven by an evidence-based comparison of the mineral bioavailability and phytochemical composition across commercial date cultivars. Relative to widely utilized commodity varieties such as Deglet Noor, Medjool demonstrates markedly superior micronutrient density: calcium concentrations reach 129.14 mg 100 g^−1^ (representing a 40–50% advantage), potassium amounts to 851.98 mg 100 g^−1^, magnesium amounts to 142.97 mg 100 g^−1^, and phosphorus amounts to 139.40 mg 100 g^−1^ [10]. The polyphenolic profile similarly distinguishes this cultivar, with its total phenolic content ranging from 452 to 554 mg GAE 100 g^−1^, positioning Medjool among the dried fruit matrices with the highest antioxidant content. The inherent carbohydrate matrix (75–80% total sugars) and dietary fiber component (6–10%) simultaneously address sweetening requirements, textural development, and prebiotic delivery without requiring refined sugar supplementation or synthetic additives [11,12]. From a food engineering perspective, date paste exhibits distinctive rheological behavior, manifesting pseudoplastic, non-Newtonian flow characteristics with elastic properties dominating the viscous response, which facilitates both processing efficiency and long-term product stability through enhanced moisture distribution, structural cohesion, and the suppression of microbial proliferation via reduced water activity (0.55–0.65) [13,14].

The polyphenolic constituent profile of Medjool dates encompasses multiple bioactive compound classes with established physiological benefits: hydroxycinnamic acids including caffeic acid (3.2–8.5 mg 100 g^−1^), ferulic acid (2.1–6.3 mg 100 g^−1^), and p-coumaric acid (1.8–4.2 mg 100 g^−1^), alongside flavanolic compounds such as catechin (1.2–3.4 mg 100 g^−1^) and epicatechin (1.0–2.9 mg 100 g^−1^), collectively substantiate its functional food classification through validated antioxidant, anti-inflammatory, and antimicrobial mechanisms [15]. Notwithstanding these advantages, the date fruit matrix exhibits a naturally low protein concentration (approximately 2.2% crude protein), creating a nutritional gap regarding contemporary consumer expectations for protein-enriched functional nutrition products. Strategic fortification utilizing milk protein concentrate (delivering 81.62% crude protein) and whey protein isolate (89.26% crude protein) resolves this limitation, enabling the developed formulations to achieve protein levels of 19.32–26.78%, with essential amino acid profiles matching or exceeding the hen’s egg reference standard for protein quality assessment [16]. This formulation integrates Medjool date paste’s minerals/polyphenols with milk proteins, boosting its macronutrients while addressing date surpluses in the Middle East and North Africa, the primary date-producing region (e.g., Egypt: 18% global production; Saudi Arabia: 16%). However, prior work has overlooked protein–polyphenol binding mechanics in date bars. Our innovation uses docking to reveal β-lactoglobulin’s stable complexes that shield phenolics from degradation, reduce browning, and ensure a firm texture, driving 85% stability gains across formulations [17].

To date, the mechanistic examination of protein–polyphenol binding dynamics within date-based bars remains unexplored. No prior studies have examined protein–polyphenol binding dynamics in date bars or linked them via multimodal analysis (SEM, DSC, colorimetry, texture) to stability/functionality. This work fills this gap, quantifying milk protein–date bioactive interactions in Medjool formulations to reveal preservation mechanisms. Therefore, this study aimed to develop high-nutrition Medjool date-based bars (MDBs) fortified with milk and plant proteins and to elucidate how such fortification influenced their functionality. Specifically, the objectives were to (i) formulate Medjool date-based bars using selected dairy and plant protein sources and (ii) evaluate their proximate composition, mineral content, amino acid profile, phytochemical constituents, texture and physical properties, protein–polyphenol interactions, and sensory acceptance.

## 2. Materials and Methods

### 2.1. Ingredients

Ingredients such as Medjool date paste, oats, almonds, peanut butter, and date syrup were purchased from Abu Auf Factory, 10th of Ramadan City, Egypt (https://www.abuauf.com). Milk protein concentrate and whey protein isolate powders were purchased from MYPROTEIN Co., Manchester, UK (www.myprotein.com). Sesame, coconut powder, Buffalo’s ghee, and wheat bran were purchased from the local market in Egypt. Salt was purchased from The Egyptian Minerals and Salts Company (EMISAL), (Fayoum, Egypt). All ingredients were analysed and illustrated in Table S1.

### 2.2. Formulation of Date-Based Bars (MDBs)

The preparation of Medjool date-based bars (MDBs) followed a modified version of the procedure described by Ibrahim et al. [11]. Raw almonds were ground using a Moulinex Odacio food processor (FP7371, Ecully, France) at speed 3 for 30 s. The dry ingredient mixture, consisting of sesame seeds, ground almonds, oat flour, milk protein concentrate powder, whey protein isolate powder, wheat bran, and salt, was roasted in an air-heated oven at 200 °C for 5 min with continuous stirring, followed by the addition of coconut powder and further roasting for 3 min at the same temperature. Simultaneously, the wet ingredients (date paste, buffalo ghee, date syrup, and peanut butter) were heated separately in the oven at 120 °C for 5 min to reduce the viscosity and improve mixing.

The proposed MDBs were formulated according to the indicated formulas in Table 1. The thermally treated dry and wet ingredients were combined in a dough mixer and mixed until a homogeneous paste was achieved. The resulting mixture was weighed to predetermined amounts using a calibrated balance and manually shaped into bars with standardized dimensions. The shaped MDBs were immediately transferred to refrigerated storage at 4 ± 1 °C and maintained in sealed containers to prevent moisture absorption and contamination until subsequent analytical evaluation or utilization. Throughout all processing phases, critical control points were monitored to ensure reproducibility and product quality, including temperature verification, timing precision, and visual assessment of ingredient uniformity.

### 2.3. Proximate Chemical Composition and Mineral Content of MDBs

The formulated MDBs were subjected to chemical analysis (moisture, crude protein, crude lipids, ash, dietary fiber, available carbohydrates) and to caloric value analysis according to the AOAC [18] methods. Total sugars, reducing sugars, and non-reducing sugars were determined according to the Lane and Eynon method No. 935.64, as given in the AOAC [18]. Sodium and potassium levels were analyzed using flame photometry (P.F.P. 7, Model Jenway 8515, Essex, UK) according to method 956.01. Calcium, magnesium, iron, copper, manganese, and zinc concentrations were measured with an atomic absorption spectrometer (Perkin-Elmer 2380, Essex, UK) in accordance with AOAC method 968.08 [18]. Phosphorus content was quantified through a standard colorimetric procedure described by Borah et al. [19].

### 2.4. Amino Acid Composition of MDBs

The amino acid compositions of the experimental samples were determined using the HPLC-Pico-Tag method, as described by Cohen et al. [20].

### 2.5. Phytochemical Analysis of MDBs

Total phenolic compounds (TPC) in MDBs were determined using the Folin–Ciocalteu method, and TPC was expressed as milligram gallic acid equivalents (mg GAE 100 g^−1^ dw), according to Bettaieb et al. [21]. Total carotenoid (TC) content was measured colorimetrically according to a modified protocol [22]. Antioxidant capacity was assessed via DPPH radical scavenging activity (DPPH-RSA), with results reported as micromoles of Trolox equivalents per gram dry weight (μmol TE g^−1^) [23]. Radical scavenging activity (ABTS-RSA) against ABTS radicals was tested using the method described by Almundarij et al. [24]. Additionally, total flavonoid (TF) and total flavonol (TFL) content were determined using methods by Barakat and Almundarij [25] and Kumaran and Karunakaran [26], respectively, with values expressed as milligrams of quercetin equivalents per gram (mg QE g^−1^).

### 2.6. Polyphenol Fractionization of MDBs

Prior to HPLC-PDA fractionation, polyphenols and flavonoids were extracted from homogenized samples (1.0 g) using acidified methanol (70% methanol–30% 0.1% formic acid in water, *v*/*v*; 10 mL) [27].

### 2.7. Polyphenol and Protein Interactions of MDBs

The molecular docking analysis investigated the binding interactions between key milk proteins (β-lactoglobulin and sodium caseinate) and epicatechin, a primary bioactive compound in date-based formulations. Three-dimensional protein structures of β-lactoglobulin and sodium caseinate were retrieved from the Research Collaboratory for Structural Bioinformatics Protein Data Bank (RCSB PDB), β-LG (PDB ID: 3NPO), and a representative casein micelle model. At the same time, the 3D geometries of epicatechin and caffeic acid were obtained from the PubChem database. Molecular docking simulations were conducted using the Discovery Studio software version 2016 to predict the binding affinities, interaction patterns, and structural conformations. Molecular docking simulations were conducted using the protein and ligand crystal structures, which were prepared by removing water molecules, adding polar hydrogens, and assigning Gasteiger charges in Discovery Studio. The docking protocol involved protein preparation, ligand optimization, binding site identification, and the comprehensive analysis of protein–ligand interactions, including binding constant calculation, 3D structural visualization, 2D interaction mapping, surface interaction analysis, and Protein–Ligand Interaction Profiler (PLIP) binding characterization to elucidate the molecular mechanisms underlying the protein–polyphenol interactions in the date bar matrices. All simulations were performed in triplicate to ensure reproducibility.

### 2.8. Instrumental Color Measurements of MDBs

The color of each sample was measured using a chromameter (ColorFlex, Reston, VA, USA) on the CIELAB scale (L*, a*, and b*) with typical white, green, and black tiles. The hue angle (H°), chroma (C), and browning index (BI) were then calculated according to [28].

### 2.9. Scanning Electron Microscopy Analysis of MDBs

The MDB samples underwent comprehensive microstructural analysis using scanning electron microscopy (SEM) and digital image processing in Origin 2025. Sample preparation followed established protocols for food materials, where specimens were carefully sectioned to expose representative cross-sectional surfaces (SU8010, Hitachi Co., Ltd., Tokyo, Japan). The samples were subsequently prepared using standard SEM preparation techniques, likely including critical-point drying or freeze-drying to preserve the native microstructure while removing moisture that could interfere with electron-beam imaging. The SEM analysis generated high-resolution surface topography images, utilizing the interaction between focused electron beams and sample atoms to produce detailed structural information. This approach enables the comprehensive characterization of surface features at magnifications ranging from 10× to over 500,000×, far exceeding the resolution limits of conventional light microscopy. The technique proves particularly valuable for food materials as it provides an exceptional depth of field and three-dimensional illustrations of surface structures.

Following SEM imaging, the acquired micrographs underwent systematic digital image processing using the Origin 2025 software, which provides robust image conversion, geometric transformation, and quantitative analysis capabilities. The image analysis workflow incorporated several critical steps: initial image acquisition, conversion to grayscale, binary thresholding for feature segmentation, and subsequent quantitative measurements of surface characteristics. The analytical approach focused on extracting quantitative parameters for surface size distribution, defect characterization, porosity analysis, and homogeneity assessment. These measurements enabled an objective comparison between samples and provided statistically robust data for microstructural evaluation. The Origin software’s advanced image processing tools enabled the precise measurement of crack dimensions, pore characteristics, and surface uniformity parameters [29].

### 2.10. Differential Scanning Calorimetry Analysis of MDBs

Differential scanning calorimetry (DSC, Shimadzu Co., Kyoto, Japan) analysis was performed on three homogenized MDB samples, with approximately 5–10 mg of each sample sealed in standard aluminum pans; an empty sealed pan served as the reference (D-8, Bruker Corp., Karlsruhe, Baden-Württemberg, Germany). The DSC instrument was calibrated with an indium standard before use. Each sample was equilibrated at 25 °C for 2 min and then heated from 25 to 200 °C at 10 °C/min under a nitrogen atmosphere (50 mL/min). Heat flow data were collected to identify thermal transitions, including onset and peak temperatures and enthalpy changes. All measurements were conducted in duplicate, and mean values were reported for analysis. The DSC constants were also calculated using OriginPro 2025, accessible at Jiangsu University [30].

### 2.11. Texture Analysis Measurements of MDBs

All samples’ texture profile analysis (TPA) was conducted using the TA.XT Plus device (Stable Micro Systems, Surrey, UK), equipped with a 50 kg load cell and a cylindrical aluminum probe with a 75 mm diameter (P/75). Samples were precisely cut into uniform cubes (20 × 20 × 20 mm^3^) using a sharp blade, conditioned at 25 °C for 2 h to standardize the temperature and moisture, and placed centrally on the analyzer base. A two-cycle compression test was performed at a pre-test speed of 2.0 mm/s, test speed of 1.0 mm/s, and post-test speed of 2.0 mm/s, with a target strain of 50% (trigger force: 5 g, pause between cycles: 5 s). Parameters including hardness (peak force during first compression), cohesiveness (ratio of positive areas: second cycle/first cycle), springiness (height recovery after first compression), chewiness (hardness × cohesiveness × springiness), and adhesiveness (negative work area during probe retraction) were derived from force–time curves using the Exponent software (v6.1). Three replicates per formulation were analyzed, and results were expressed as the mean ± standard deviation [31].

### 2.12. Sensory Evaluation of MDBs

To conduct the sensory evaluation of the final formulation, 12 well-trained participants (21–58 years) from the Faculty of Agriculture at Benha University evaluated the date bars. A composite scale was used to assess the taste, appearance, aroma, color, and texture of the date bars. Using this scale, we determined the level of trained panel evaluation and satisfaction. The information collected during sensory evaluations is helpful in product development, quality control, and marketing strategies [32].

### 2.13. Statistical Analysis

Statistical analysis was performed using SPSS (Ver. 27.0 for Windows, IBM, Armonk, NY, USA). Statistical significance was tested with a one-way ANOVA followed by Tukey’s post hoc test, and *p*-values < 0.05 were considered significant, according to Steel et al. [33].

## 3. Results and Discussion

### 3.1. Proximate Chemical Composition of Formulated MDBs

The nutritional characterization of the three Medjool date-based bar formulations (designated T1, T2, and T3) revealed statistically differentiated compositional profiles that aligned with the escalating protein fortification strategy employed in the formulation design (Table 2). Multivariate analysis confirmed significant compositional variations across formulations in five nutrient parameters: moisture, crude protein, mineral ash, available carbohydrates, and sucrose content (*p* < 0.05). The moisture content, regulated primarily by the hydrophilic properties of the date paste matrix and the inherent moisture-binding capacity of the ingredient system, demonstrated limited variation (T1: 12.71%; T2: 12.46%; T3: 12.35%), with statistical significance observed only between T1 and T3. The constrained moisture range reflects effective formulation control. It indicates the robust preservation of textural properties despite protein-level variation, consistent with the literature documenting moisture-dependent texture stability in protein-enriched cereal matrices [6]. The crude protein content, conversely, exhibited substantial differentiation, reflecting the deliberate protein fortification hierarchy: T1 (19.32%), T2 (23.20%), and T3 (26.78%) represent progressive enrichment from baseline to premium positioning. This 7.46% increase in point differential spans high-protein category thresholds (≥20% at T2/T3 formulations) to premium sports nutrition density (≥25% at T3), aligning with commercial product positioning across diverse consumer segments. The T3 formulation achieves a protein concentration that is substantially higher than previously published date-based snack bar formulations by 4–8 percentage points, substantiating the functional innovation of protein-fortified date matrices. The mineral profile, reflected in ash content determination, increased concordantly with protein fortification (T1: 2.87%; T2: 3.14%; T3: 3.27%), indicating a proportional increase in micronutrient density from the mineral-enriched protein concentrates (milk protein concentrate: 0.35% ash; whey protein isolate: 0.32% ash). The sucrose concentration exhibited formulation-dependent variation (T1: 2.05%; T2: 2.06%; T3: 2.10%), with a statistically significant elevation in T3, attributable to higher date paste inclusion relative to the total formula weight [34]. Notably, the lipid composition (10.96% across all formulations), dietary fiber (6.42–7.15%), total sugars (36.77–36.98%), and reducing sugar profiles (34.60–34.93%) remained statistically invariant, indicating that base ingredient contributions (date paste, nuts, seeds) dominated these nutrient fractions, rather than the protein fortification level. The stability of the energy density across formulations (T1: 414.72 Kcal/100 g; T2: 415.20 Kcal 100 g^−1^; T3: 416.04 Kcal 100 g^−1^) represents a critical formulation achievement, maintaining caloric equivalence despite substantial macronutrient redistribution through compensatory reductions in available carbohydrate content (T1: 59.70%; T2: 55.95%; T3: 52.57%). This energy stability, consistent with the principles of macronutrient balance in functional food design, enables consumer choice based on protein objectives without altering portion-size caloric expectations—a commercially significant advantage enabling cross-product positioning and co-merchandising strategies [35].

### 3.2. Mineral Content of MDBs

The mineral content of the MDBs is tabulated in Table 3. A significant difference (*p* < 0.05) was found among treatments for all elements. The mineral profiles of MDBs demonstrated progressive enrichment from T1 to T3. T3 contained the highest content of Na, K, Ca, Mg, P, Cu, Zn, and Mn, significantly reflecting cumulative contributions from escalating protein fortification. T1 contained the lowest content of all elements except Se and Fe, while it contained 34.11 and 2.96 mg 100 g^−1^ Se and Fe, respectively. Se levels remained statistically invariant across formulations, while Fe concentrations declined significantly from T1 to T3. T2 contained moderate levels of mineral elements compared to the other two treatments. The observed patterns position the T3 formulation as a mineral-dense functional food. These results are higher than those obtained in [36] for most minerals, except Fe, Zn, and Mn, which were comparable.

Regarding the evaluation of the mineral content in MDBs, it is apparent that MDBs contain appreciable amounts of Ca, Mg, P, Fe, Cu, and Zn compared with many other foods. MDBs are rich sources of Ca (average content of 501.76 mg 100 g^−1^)—even richer than cow’s milk, which has been reported to contain 72 mg 100 g^−1^ [37]. The average concentration of Mg in MDBs (175.61 mg/100 g) is much higher than that found in human milk (4 mg 100 mL^−1^) or cow’s milk (12 mg 100 mL^−1^) [38]. The amount of P present in MDBs (average content of 430.99 mg/100 g) is higher than that found in dry milk (250 mg 100 g^−1^) and comparable to that found in meat and meat products (392 to 499 mg 100 g^−1^) [37,39]. With an average value of about 2.72 mg/100 g of iron, it contains one third to one half of that present in the liver and kidney, which are reported to contain 8 and 6 mg/100 g [39]. With an average value of 0.83 mg 100 g^−1^, MDBs are good sources of Cu compared to human milk (0.6 to 1.05 mg L^−1^), as reported in [40]. Considering that Zn is becoming more deficient in human foods [38], the average value of 3.14 mg 100 g^−1^ indicates that MDBs could be regarded as a richer source than milk (3–5 µg g^−1^) or even seafood (15 µg g^−1^) [40]. The measured mineral content of MDBs reveals their potential to meet the recommended dietary allowance and, in turn, the nutritional requirements of humans for most minerals.

### 3.3. Amino Acid Composition of MDBs

MDBs were characterized for their amino acid compositions (Table 4). The results showed 17 identified amino acids, with clear enrichment in essential amino acids (EAAs), in line with the increasing protein fortification across treatments. T3 displayed the highest EAA content, followed by T2, while T1 had the lowest; in T2, the levels of several EAAs (threonine, valine, methionine, leucine, lysine, and histidine) matched or exceeded those of the hen’s egg reference. In T3, all EAAs except cysteine and tyrosine reached egg protein equivalence [41]. Branched-chain amino acids (leucine, isoleucine, valine) represented the most abundant EAAs in all formulations, with T3 values approaching those targeted in sports nutrition products [42], and lysine and methionine similarly achieved egg protein comparability, thereby indicating complete protein functionality [43]. Non-essential amino acids showed formulation-dependent variation, reflecting contributions from both Medjool date matrices and protein concentrates, but the overall amino acid balance was most favorable in T3, which combined a high EAA density with reduced non-essential fractions. Collectively, these findings confirm that the fortified MDBs, particularly T2 and T3, can be regarded as high-quality, near-complete protein sources that meet or surpass the hen’s egg reference standard [44].

### 3.4. Phytochemical Analysis of MDBs

The phytochemical profiles, including TPC, TF, TFL, DPPH-RSA, and ABTS-RSA, were determined in the formulated MDBs, and the results are presented in Table 5. TPC and TF decreased significantly with increasing protein fortification, with T3 showing the lowest values, while T1 and T2 did not differ significantly from each other. TFL also declined from T1 to T3, but this trend was not statistically significant. In contrast, the antioxidant activity (DPPH and ABTS assays) and TC remained statistically unchanged across treatments, indicating that the radical scavenging capacity was preserved despite reductions in some individual phenolic fractions. In summary, a decrease in total phenolic and flavonoid content was observed with increasing treatment levels. In contrast, the antioxidant capacity (DPPH and ABTS) and total carotenoids remained statistically unchanged, suggesting the stability of the radical scavenging potential despite reductions in some individual phytochemicals.

The formulated Medjool date bars showed differential phytochemical stability. TPC declined by 18.4% (554.12 to 452.22 mg GAE 100 g^−1^) through thermal degradation, while flavonoids remained stable (4.3% decrease), reflecting the thermal resistance of date flavonol glycosides. TFL decreased non-significantly (279.15 to 244.77 mg QE 100 g^−1^), suggesting conversion to aglycones rather than complete loss [22,46,47].

Despite phenolic reductions, DPPH (395.59–398.36 mmol TE 100 g^−1^) and ABTS (362.27–376.16 mmol TE 100 g^−1^) activity remained unchanged, reflecting mechanistic compensation: residual phenolic acids maintain efficient radical scavenging despite lower concentrations; carotenoids provide complementary free radical trapping through independent pathways; and synergistic phenolic–carotenoid interactions buffer the overall antioxidant capacity [48,49]. The total carotenoid content showed a non-significant decline (345.80 to 308.97 µg 100 g^−1^, ~10.6%). This modest change is noteworthy given carotenoids’ inherent oxidative lability, reflecting the protection provided by the date bar matrix, its high sugar content, lipophilic ingredients, and residual polyphenolics that sequester reactive oxygen species and limit free radical-mediated carotenoid cleavage [50]. The preservation of the radical scavenging activity despite reduced phenolic content demonstrates that the antioxidant capacity in complex matrices reflects multifactorial compensation. Stable DPPH/ABTS values indicate sufficient coverage through stable phenolic acids, distinct carotenoid pathways, and multicomponent interactions, emphasizing the importance of measuring both the composition and biological activity [51].

### 3.5. Polyphenol Analysis of MDBs

**Figure 1:**
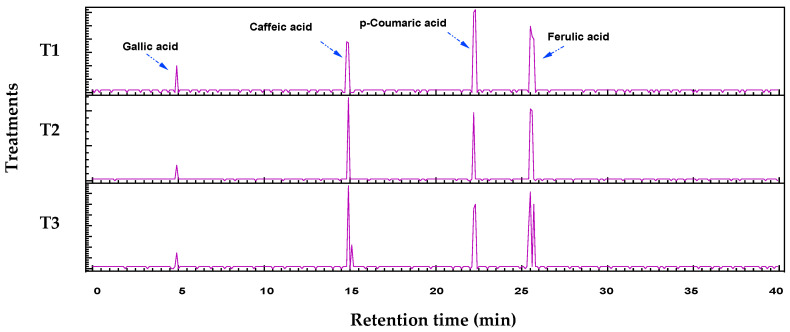
Fractionalization of phenolic acids, flavonoids, and other existing polyphenols in the three treatments prepared. The peak area of each polyphenol is tabulated in Table 6.

The chromatograms and data tables illustrate the polyphenol profiles for the three protein bar formulations (T1, T2, and T3) obtained by HPLC-PDA. The retention times were calculated for specific compounds (e.g., gallic acid ~5 min, catechin ~10 min, caffeic acid ~15 min, epicatechin ~18 min, p-coumaric acid ~22 min, ferulic acid ~25 min, quercetin ~35 min). At the same time, peak areas indicate relative concentrations, enabling comparisons of formulation effects on polyphenol diversity and abundance (Figure 1 and Table 6).

The HPLC-PDA chromatographic analysis revealed the comprehensive polyphenolic profile across the protein bar samples, with excellent compound separation achieved over a 40 min gradient. Eight major polyphenolic compounds were successfully identified and quantified, including phenolic acids (gallic acid, caffeic acid, p-coumaric acid, ferulic acid) and flavonoids (catechin, epicatechin, rutin, quercetin), with retention times ranging from 5 to 34 min. This polyphenol composition comprises plant-derived ingredients commonly used in protein bar formulations, including grains, nuts, seeds, and fruit extracts. Significant compositional differences were observed among the analyzed samples. T1, T2, and T3 demonstrated similar polyphenolic profiles characterized by high concentrations of hydroxycinnamic acids, with caffeic acid showing the highest abundance (1908.36–4873.5 units), followed by p-coumaric acid (2858.436–4001.4 units) and ferulic acid (2544.48–4216.86 units). Gallic acid was present in moderate concentrations (759.24–1026 units) across these three treatments. Indeed, natural source-derived phenolic acids exhibit potent cardioprotective and anti-inflammatory effects by neutralizing oxidative stress, inhibiting NF-κB/MAPK pathways, and reducing proinflammatory cytokines while enhancing vascular function [16]. The presence of these compounds enhances the functional food value of the protein bars beyond their primary nutritional contribution of protein content.

### 3.6. Protein and Polyphenol Interactions of MDBs

**Figure 2:**
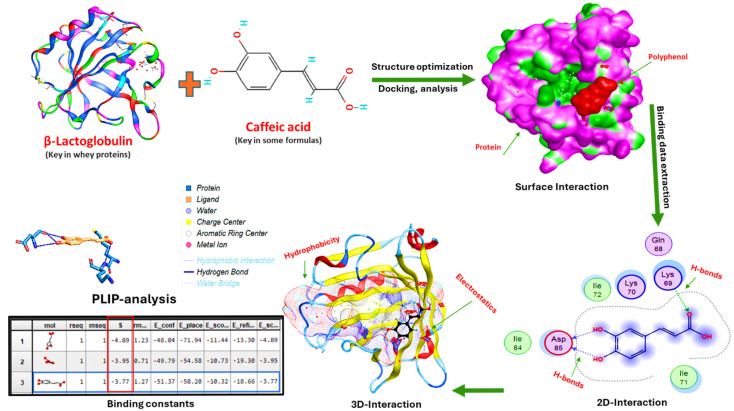

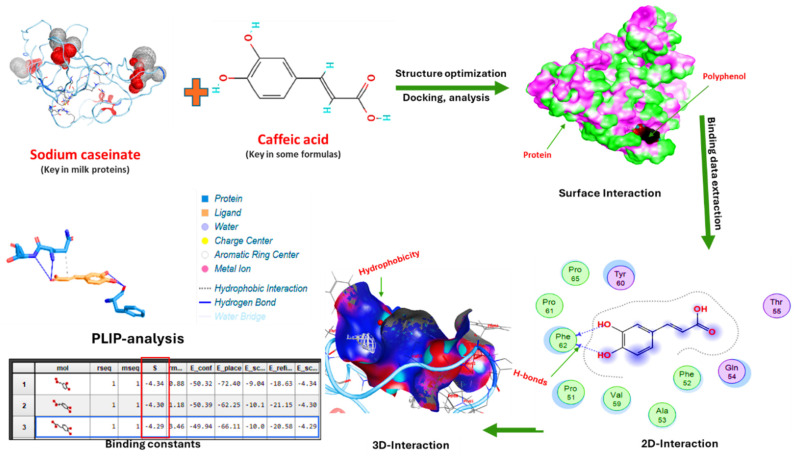
The interactions between β-lactoglobulin (key protein in whey) and/or sodium caseinate (key in milk proteins) and caffeic acid (key in some formulas), as well as their binding constants, 3D structures, 2D interactions, surface interactions, and PLIP binding analysis.

Molecular docking analysis revealed distinct binding mechanisms between caffeic acid and the milk proteins examined, with significant implications for the date bar formulations. β-Lactoglobulin demonstrated strong binding affinity (ΔG = −71.94 kJ/mol), with caffeic acid successfully penetrating the hydrophobic binding pocket through multiple hydrogen bonds with key amino acid residues (Gln68, Lys69, Asp85, Ile84) and aromatic π–π stacking interactions; see Figure 2. The molecular docking of milk proteins with caffeic acid, showing strong binding interactions that form stable complexes against oxidative degradation, reduces protein aggregation during processing and enhances overall protein solubility [52].

Sodium caseinate exhibited distinct binding characteristics, with moderate affinity (ΔG = −58.32 kJ/mol), and caffeic acid interacted through a more flexible binding region involving proline-rich residues (Pro65, Pro81, Pro62) and aromatic amino acids (Tyr60, Phe52). This flexible interaction pattern utilizes both hydrophobic and hydrophilic regions of the protein, suggesting a versatile binding mechanism that enables gradual polyphenol release during digestion, potentially improving bioavailability [53].

These complementary protein–polyphenol interaction patterns have important technological implications. The strong binding of β-lactoglobulin provides robust antioxidant protection and contributes to protein network formation. At the same time, sodium caseinate’s moderate affinity enhances its emulsification and gel-forming properties while modulating the rheological properties. Together, these interactions strengthen the date bar matrix by enhancing protein–protein and protein–polyphenol cross-linking, improving the water-holding capacity, and enhancing texture stability. The differential binding profiles suggest that strategic protein blending could optimize formulation performance, thereby maximizing both the nutritional delivery of bioactive compounds and the functional properties, producing date bars with superior shelf stability, sensory qualities, and health-promoting potential [54,55].

The molecular docking analysis revealed distinct binding modes for caffeic acid with the two milk proteins examined, with important implications for polyphenolic stabilization in date bar formulations. β-Lactoglobulin demonstrated strong binding affinity (ΔG = −71.94 kJ/mol), with caffeic acid penetrating the hydrophobic calyx pocket through hydrogen bonds with key amino residues (Gln68, Lys69, Asp85, Ile84) and π–π stacking interactions. This binding pattern aligns with the established literature, in which caffeic acid exhibits static quenching and multiple van der Waals and hydrogen bonding interactions with whey proteins. The thermodynamically stable complex effectively sequesters caffeic acid from oxidative degradation during processing and storage, reducing enzymatic browning via polyphenol oxidase pathways while enhancing protein solubility and preventing aggregation—critical for formulated bar matrices [47,48].

Sodium caseinate exhibited moderate binding affinity (ΔG = −58.32 kJ/mol) through flexible, multi-site regions involving proline-rich residues (Pro65, Pro81, Pro62) and aromatic amino acids (Tyr60, Phe52), reflecting caseinate’s intrinsically disordered structure. This versatile binding enables gradual polyphenol release during digestion, enhancing bioavailability compared to high-affinity binding, which impedes digestive enzyme access. Strategic protein blending combines β-lactoglobulin’s strong protective binding for shelf stability with caseinate’s moderate affinity for controlled bioavailability, creating complementary mechanisms that enhance protein–polyphenol cross-linking, improve the water-holding capacity, strengthen the product matrix, and optimize both the functional and nutritional properties in shelf-stable date bars [56].

### 3.7. Visual Instrumental Colors of MDBs

Table 7 presents the colorimetric parameters for the T1–T3 formulations. Lightness (L*) rose significantly from T1 to T3, while a* (redness) dropped markedly from T1 to T2 (no further change to T3); b* (yellowness) remained steady across treatments. Chroma (C*) increased from T2 to T3; the b*/a* ratio rose from T1 to T3; the hue angle (H°) declined from T1 to T2/T3 levels; the browning index (BI) fell sharply from T1 to T3; and the color difference (ΔE vs. T1) grew significantly in T2 and T3. The Medjool date bars showed browning suppression with advancing protein fortification: higher L*, lower a* and BIs, stable b*, a hue shift to yellower tones, and a perceptible ΔE, driven by polyphenol oxidase inhibition and Maillard reaction limitation via protein cross-linking, yielding lighter, fresher hues for better shelf-life and appeal [57,58].

### 3.8. Microstructural Analysis of MDBs

**Figure 3:**
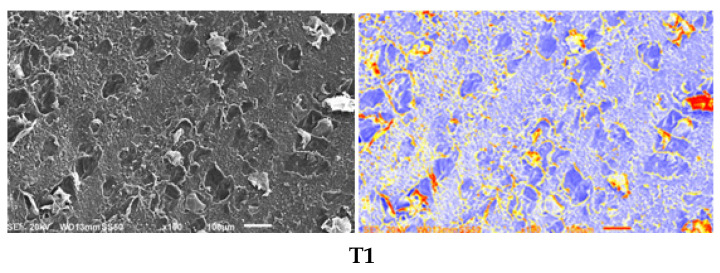

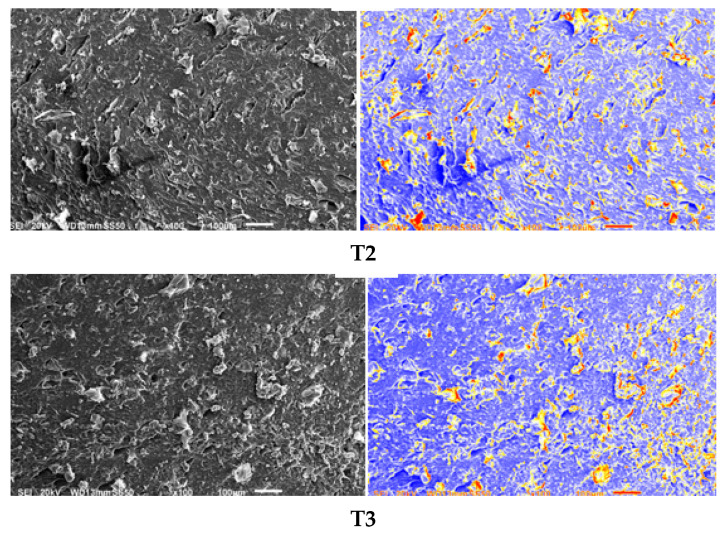
Scanning electron microscopy for the three treatments, as well as their image analysis using Origin 2025. The method is fully explained in the Materials and Methods section.

SEM analysis combined with digital image processing revealed significant microstructural variations among the three formulations tested (Figure 3). The samples demonstrated distinct patterns in surface topology, defect density, and material homogeneity that correlated with their processing parameters and ingredient compositions [59]. T2 showed the most pronounced structural heterogeneity, with distinct regions of differing material characteristics, suggesting incomplete mixing or phase separation. Extensive yellow and red coding in processed images, multiple crack-like features, and increased porosity were observed, indicating substantial spatial variations that could compromise product quality and shelf stability [60]. T1 and T3 showed balanced microstructures with fair homogeneity, moderate roughness/porosity (T1), and even defect distribution (T3). Sample T2 had significant heterogeneity, and the flaws likely stem from poor mixing or stress buildup, which could compromise its strength and shelf life. SEM image analysis reliably quantified these traits (homogeneity, defects, porosity), proving valuable in fine-tuning the quality of hydrocolloid confectionery and its processes [34]. This approach provides critical insights for refining formulations and processing to achieve desired microstructural outcomes that meet commercial standards.

### 3.9. Differential Scanning Calorimetry Analysis of MDBs

**Figure 4:**
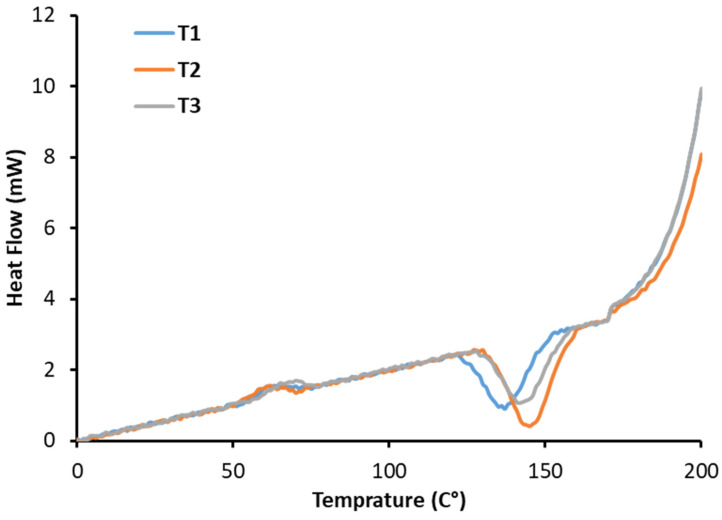
Differential scanning calorimetry analysis of the three treatments prepared, as well as the control. The DSC constants are tabulated in Table 8.

Figure 4 and Table 8 present the DSC results for the three treatments prepared and their thermal analysis constants. The differential scanning calorimetry analysis revealed significant variations in glass transition temperatures across the MDB formulations, ranging from 143 to 147 °C, which directly correlated with their structural integrity and storage stability. For example, T1 exhibited the highest Tg at 147 °C, reflecting a rigid amorphous matrix with restricted molecular motion due to stronger interactions [61]. Meanwhile, T2 showed identical Tg values of 143 °C across different formulations, suggesting similar amorphous network structures, whereas T3 displayed an intermediate Tg of 145 °C. These differences in glass transition temperature have profound implications for textural properties and thermal stability, as higher Tg samples maintain structural integrity longer under ambient conditions and exhibit superior resistance to temperature-induced textural changes during storage and distribution [17].

On the other hand, the enthalpy values revealed dramatic differences in thermal transition energies, with T1 exhibiting exceptionally high enthalpy at 11.81 J/g, indicating substantial crystalline regions or organized molecular structures that require significant energy disruption. Conversely, T2 demonstrated remarkably low enthalpy of 0.46 J/g despite its high Tg (143 °C), creating a unique structural profile of restricted molecular mobility within a predominantly amorphous matrix, whereas T3 (4.18 J/g) showed intermediate enthalpy values consistent with its structural organization. The combination of a high glass transition temperature and elevated enthalpy in T1 suggests optimal structural integrity for MDB applications, providing both thermal stability and an organized molecular architecture that translates into superior texture retention and processing characteristics. These DSC results provide essential foundation data for production scale-up, the prediction of shelf-life performance, and the optimization of processing parameters to achieve consistent product quality across MDB product lines [62].

### 3.10. Texture Analysis of MDBs

The texture profile analysis (TPA) of the three treatments reveals significant variations in key mechanical properties, which directly influence sensory perception and consumer acceptability (Table 9). Higher hardness values (e.g., T1) suggest denser or more cross-linked structures, potentially due to protein aggregation or reduced moisture content. In contrast, lower hardness (T2) may reflect softer matrices, possibly influenced by plasticizing ingredients like humectants or fats [63]. Cohesiveness varies from 0.74 (T2). T2’s high cohesiveness implies strong internal bonding, likely to enhance structural integrity. Springiness reaches 0.67 (T1), where higher values (T2 and T3) suggest greater elasticity, potentially from polymeric protein networks or hydrocolloids. Chewiness correlates with the product of hardness, cohesiveness, and springiness. T2 (95.47) exhibits the highest chewiness, requiring more oral processing, whereas T1 (56.87) was less demanding. This aligns with studies linking high chewiness to increased protein content or fiber inclusion [64]. Regarding adhesiveness, all samples show negative values (indicating adhesive forces), ranging from −0.23 (T1) to −0.52 (T2). T2’s pronounced adhesiveness may result from hydrophilic ingredients such as syrups or proteins, potentially leading to undesirable mouth-coating. These variations likely stem from differences in formulation (e.g., protein type, moisture, sweeteners) or processing (e.g., mixing intensity, curing time). For instance, high hardness/low cohesiveness combinations can lead to perceived dryness, while balanced profiles (e.g., T3) may optimize palatability [65]. Future work should correlate these parameters with sensory panels to define ideal textural benchmarks for consumer acceptance.

### 3.11. Sensory Evaluation of MDBs

The data recorded in Table 10 show the sensory evaluation results for treatments T1, T2, and T3. Parameters such as appearance, aroma, taste, texture, and overall acceptability were found to be statistically similar across treatments, indicating that treatment differences did not significantly influence panelists’ perceptions. Appearance scores remained steady (T1: 18.27–T2: 19.00). Aroma showed minor, non-significant variation (T2 lowest at 17.36). Color differed significantly (T2/T3: 18.73 > T1: 16.73). Taste rose from 17.00 (T1) to 18.64 (T3), and texture rose from 17.36 to 18.55, both being non-significant. Overall liking increased from 87.63 (T1) to 93.28 (T3), confirming the strong acceptance across formulations with enhanced color and taste.

The sensory evaluation revealed generally high acceptance among panelists across all treatments, with appearance and aroma remaining consistent, reflecting stable fundamental sensory characteristics despite formulation modifications. Color emerged as the only statistically significant parameter, with T2 and T3 scoring higher than T1. This is mechanistically consistent with the instrumental colorimetric analysis, showing reduced browning and improved hue angles in higher treatments—improvements that consumers associated with perceived freshness and premium quality [66]. Taste and texture scores trended upward, although not significantly, suggesting that panelists perceived incremental improvements aligned with protein–polyphenol interactions that enhanced products’ rheology and mouthfeel. Overall acceptability demonstrated the most pronounced treatment effect, rising significantly from 87.63 (T1) to 93.28 (T3), corresponding to “like very much” hedonic equivalents and indicating commercial viability [67]. This progressive improvement in overall acceptability, coupled with color enhancement and maintained sensory integrity in appearance and aroma, validates the formulation optimization and demonstrates successful alignment between functional benefits, improved antioxidant stability, enhanced protein–polyphenol networks, and consumer sensory preferences—a level of concordance that predicts commercial market success [11]. These uniform results from trained panelists indicate that the protein fortification levels produced comparable sensory profiles, with only the brighter color in T2/T3 aligning with instrumental reductions in browning, suggesting maintained quality across formulations.

## 4. Conclusions

This research formulated a nutrient-dense, protein-enriched Medjool date bar enhanced with milk protein concentrate, whey protein isolate, nuts, seeds, and bioactive additives. The optimized formulation achieved protein digestibility equivalent to established benchmarks; delivered substantial calcium, phosphorus, and zinc at levels comparable to traditional sources; and maintained robust antioxidant capacity from hydroxycinnamic acids, contributing to potential anti-inflammatory and cardiovascular benefits. Milk protein–polyphenol interactions facilitated improved bioavailability while providing protective encapsulation during processing and storage. Thermal and structural analysis demonstrated appropriate glass transition behavior and mechanical properties aligned with panelists’ expectations; sensory evaluation confirmed high satisfaction across formulations. The investigation reveals the commercial feasibility of date bars as competitive functional snacks, distinguished by superior amino acid profiles, sustained antioxidant activity, and enhanced mineral bioavailability. Future research should prioritize market readiness through optimized moisture barrier packaging for shelf-life extension, manufacturing scalability assessment, and consumer acceptance studies across demographic groups to validate their commercial viability and advance date palm utilization in regional food systems.

## Figures and Tables

**Table 1:** Edible ingredients of formulated MDBs (g 100 g^−1^).

Ingredient	MDB Formula g 100 g^−1^		
	T1	T2	T3
Date paste	50	50	50
Milk protein con.	7.5	10	12.5
Whey protein isolate	7.5	10	12.5
Oat	10	5	0
Almond	5	5	5
Date syrup	4.5	4.5	4.5
Sesame	4	4	4
Buffalo’s ghee	4	4	4
Coconut powder	3	3	3
Wheat bran	2	2	2
Peanut butter	2	2	2
Salt	0.5	0.5	0.5
Total mass (g)	100	100	100

**Table 2:** Proximate chemical compositions of MDBs (mean ± SE), *n* = 6.

Component	Treatment		
	T1	T2	T3
Moisture (%)	12.71 ± 0.15 ^a^	12.46 ± 0.03 ^ab^	12.35 ± 0.03 ^b^
Crude protein (%) *	19.32 ± 0.18 ^c^	23.20 ± 0.41 ^b^	26.78 ± 0.96 ^a^
Ether extract (%) *	10.96 ± 0.38 ^a^	10.96 ± 0.53 ^a^	10.96 ± 0.20 ^a^
Ash (%) *	2.87 ± 0.04 ^b^	3.14 ± 0.05 ^a^	3.27 ± 0.12 ^a^
Crude fiber (%) *	7.15 ± 0.47 ^a^	6.76 ± 0.02 ^a^	6.42 ± 0.14 ^a^
Available carbohydrates (%) ^@^*	59.70 ± 0.15 ^a^	55.95 ± 0.69 ^b^	52.57 ± 1.31 ^c^
Total sugars (%) *	36.98 ± 0.24 ^a^	36.77 ± 0.14 ^a^	36.86 ± 0.15 ^a^
Reducing sugars (%) *	34.93 ± 0.10 ^a^	34.60 ± 0.09 ^a^	34.65 ± 0.11 ^a^
Non-reducing sugars (%) *	2.05 ± 0.01 ^b^	2.06 ± 0.01 ^b^	2.10 ± 0.01 ^a^
Energy (calorie/100 g) *	414.72 ± 3.95 ^a^	415.20 ± 2.50 ^a^	416.04 ± 0.42 ^a^

^@^: Calculated by differences. *: On dry weight basis. ^a,b,c^: There is no significant difference (*p* > 0.05) between any two means within the same row having the same superscript letter.

**Table 3:** Mineral content of formulated MDBs (mg 100 g^−1^) (mean ± SE), *n* = 6.

Mineral	Treatment		
	T1	T2	T3
Na	75.96 ± 0.55 ^c^	95.69 ± 0.69 ^b^	115.42 ± 0.83 ^a^
K	884.28 ± 6.39 ^b^	904.08 ± 6.53 ^ab^	923.89 ± 6.67 ^a^
Ca	418.06 ± 3.02 ^c^	501.76 ± 3.63 ^b^	585.45 ± 4.23 ^a^
Mg	170.64 ± 1.23 ^c^	175.62 ± 1.27 ^b^	180.58 ± 1.3 ^a^
P	402.27 ± 2.91 ^c^	430.99 ± 3.11 ^b^	459.71 ± 3.32 ^a^
Cu	0.81 ± 0.01 ^c^	0.83 ± 0.01 ^b^	0.85 ± 0.01 ^a^
Zn	2.99 ± 0.02 ^c^	3.14 ± 0.02 ^b^	3.30 ± 0.02 ^a^
Mn	1.62 ± 0.01 ^c^	1.42 ± 0.01 ^b^	1.23 ± 0.01 ^a^
Se	34.11 ± 0.25 ^a^	33.96 ± 0.24 ^a^	33.81 ± 0.24 ^a^
Fe	2.98 ± 0.02 ^a^	2.72 ± 0.02 ^b^	2.46 ± 0.02 ^c^

^a,b,c^: There is no significant difference (*p* > 0.05) between any two means within the same row having the same superscript letter.

**Table 4:** Amino acid compositions of MDBs (g g^−1^ nitrogen).

Amino Acid	Treatment *			Hen’s Egg (FAO, 1970) [45]
	T1	T2	T3	
Threonine	0.321	0.322	0.325	0.320
Valine	0.399	0.429	0.438	0.428
Methionine	0.209	0.216	0.213	0.210
Cysteine	0.101	0.106	0.107	0.110
Isoleucine	0.382	0.391	0.394	0.393
Leucine	0.554	0.561	0.569	0.551
Tyrosine	0.228	0.218	0.237	0.260
Phenylalanine	0.354	0.353	0.366	0.358
Lysine	0.445	0.437	0.442	0.436
Histidine	0.149	0.154	0.157	0.152
Aspartic	0.494	0.605	0.569	0.601
Glutamic	0.844	0.864	0.855	0.796
Serine	0.370	0.377	0.344	0.478
Proline	0.381	0.285	0.234	0.260
Glycine	0.253	0.249	0.261	0.207
Alanine	0.287	0.214	0.302	0.370
Arginine	0.347	0.347	0.351	0.381
Total EAAs	3.142	3.187	3.248	3.218
Total N-EAAs	2.978	2.938	2.914	3.093
Total amino acids	6.118	6.128	6.164	6.311

*: mean of duplicate analysis, EAA: essential amino acid, N-EAA: non-essential amino acid.

**Table 5:** Phytochemical content of MDBs (mean ± SE), *n* = 6.

Parameter	Treatment		
	T1	T2	T3
TPC (mg GAE 100 g^−1^)	554.12 ± 26.82 ^a^	520.18 ± 17.17 ^ab^	452.22 ± 19.47 ^b^
TF (mg QE 100 g^−1^)	374.24 ± 2.39 ^a^	365.51 ± 3.44 ^ab^	358.06 ± 1.89 ^b^
TFL (mg QE 100 g^−1^)	279.15 ± 7.73 ^a^	255.47 ± 8.84 ^a^	244.77 ± 17.57 ^a^
DPPH (m mol TE 100 g^−1^)	396.80 ± 15.27 ^a^	395.59 ± 9.89 ^a^	398.36 ± 25.43 ^a^
ABTS (m mol TE 100 g^−1^)	362.27 ± 22.14 ^a^	367.08 ± 32.69 ^a^	376.16 ± 26.25 ^a^
TC (µg 100 g^−1^)	345.80 ± 17.65 ^a^	318.67 ± 17.61 ^a^	308.97 ± 28.82 ^a^

^a,b^: There is no significant difference (*p* > 0.05) between any two means within the same row having the same superscript letter.

**Table 6:** The intensity of the peak area of each identified polyphenol in the three treatments prepared.

Parameter	T1	T2	T3
Gallic acid (RT: 5 min)	1026	913.14	759.24
Catechin (RT: 10 min)	--	--	--
Caffeic acid (RT: 15 min)	1908.36	4873.5	3965.49
Epicatechin (RT: 18 min)	--	--	--
p-Coumaric acid (RT: 22 min)	3078	4001.4	2858.436
Ferulic acid (RT: 25 min)	2544.48	4216.86	3673.08
Rutin (RT: 28 min)	--	--	--
Quercetin (RT: 34 min)	--	--	--

--: Not detected.

**Table 7:** Visual instrumental colors of MDBs (mean ± SE), *n* = 6.

Parameter	T1	T2	T3
L*	49.14 ± 0.22 ^c^	50.78 ± 0.70 ^b^	52.78 ± 0.16 ^a^
a*	7.44 ± 0.24 ^a^	6.55 ± 0.14 ^b^	6.76 ± 0.10 ^b^
b*	25.05 ± 0.13 ^a^	24.92 ± 0.46 ^a^	25.9 ± 0.05 ^a^
C	26.14 ± 0.12 ^ab^	25.77 ± 0.41 ^b^	26.77 ± 0.04 ^a^
b/a	3.37 ± 0.11 ^b^	3.81 ± 0.15 ^a^	3.83 ± 0.06 ^a^
H°	253.50 ± 0.53 ^a^	75.29 ± 0.54 ^b^	75.41 ± 0.22 ^b^
BI	80.15 ± 0.63 ^a^	74.94 ± 0.07 ^b^	74.87 ± 0.25 ^b^
ΔE	0 ± 0 ^c^	2.09 ± 0.67 ^b^	3.99 ± 0.14 ^a^

L*: lightness (0 = black, 100 = white); a*: red (+)/green (−); b*: yellow (+)/blue (−); C: chroma; H°: hue angle; b/a: yellowness/redness ratio; BI: browning index; ΔE: total color difference. ^a,b,c^: There is no significant difference (*p* > 0.05) between any two means within the same row having the same superscript letter.

**Table 8:** Differential scanning calorimetry constants for the three treatments prepared and the control.

Parameter	Treatment		
	T1	T2	T3
Glass Transition Temperature (Tg, °C)	147.0	143.0	145.0
Enthalpy (ΔH, J/g)	11.81	0.46	4.18

**Table 9:** The textural analysis parameters for the three treatments prepared.

Parameter	Treatment		
	T1	T2	T3
Hardness	188.73 ± 2.39 ^a^	157.13 ± 2.26 ^c^	175.17 ± 2.22 ^b^
Cohesiveness	0.45 ± 0.01 ^c^	0.74 ± 0.01 ^a^	0.56 ± 0.01 ^b^
Springiness	0.67 ± 0.01 ^c^	0.82 ± 0.01 ^b^	0.88 ± 0.01 ^a^
Chewiness	56.87 ± 1.76 ^c^	95.47 ± 1.14 ^a^	86.10 ± 2.48 ^b^
Adhesiveness	−0.23 ± 0.01 ^a^	−0.52 ± 0.01 ^c^	−0.38 ± 0.01 ^b^

^a,b,c^: There is no significant difference (*p* > 0.05) between any two means within the same row having the same superscript letter.

**Table 10:** Sensory evaluation of MDBs (mean ± SE), *n* = 12.

Parameter	Treatment		
	T1	T2	T3
Appearance	18.27 ± 0.51 ^a^	19.00 ± 0.33 ^a^	18.82 ± 0.38 ^a^
Aroma	18.27 ± 0.51 ^a^	17.36 ± 0.92 ^a^	18.82 ± 0.40 ^a^
Color	16.73 ± 0.80 ^b^	18.73 ± 0.36 ^a^	18.73 ± 0.41 ^a^
Taste	17.00 ± 1.23 ^a^	18.18 ± 0.46 ^a^	18.64 ± 0.39 ^a^
Texture	17.36 ± 0.87 ^a^	18.55 ± 0.47 ^a^	18.27 ± 0.49 ^a^
Overall acceptability	87.63 ± 2.89 ^a^	91.82 ± 1.88 ^a^	93.28 ± 1.56 ^a^

^a,b^: There is no significant difference (*p* > 0.05) between any two means within the same row having the same superscript letter.

## Data Availability

The original contributions presented in this study are included in the article/Supplementary Materials. Further inquiries can be directed to the corresponding author.

## Supplementary Materials

The following supporting information can be downloaded at: https://www.mdpi.com/article/10.3390/foods15050887/s1, Table S1: Proximate chemical composition of raw materials (g 100 g^−1^ on wet weight basis), (mean ± SE), *n* = 6.
