# Supplementary material for: Formulation and Characterization of Nutrient-Dense Medjool Date Bars: Integration of Proteins, Bioactives, and Processing Stability for Functional Snack Innovation
Source: Foods. 2026 Mar 5;15(5):887. doi: 10.3390/foods15050887 (PMC12984689; doi:10.3390/foods15050887)
Supplement: Supplementary file 1 [file foods-15-00887-s001.zip › foods-4160500-supplementary.pdf]

**Table S1. Proximate chemical composition of raw materials (g 100 $g^{-1}$ on wet weight basis), (mean±SE), *n*=6**

| Raw material | Components (%) | | | | | |
|---|---|---|---|---|---|---|
| | Moisture | Crude protein | Ether extract | Ash | Crude fiber | Available carbohydrate@ |
| Date paste | 12.86 ±1.46 | 2.22 ±0.28 | 1.08 ±0.08 | 2.26 ±0.28 | 5.14 ±0.96 | 76.44 ±2.53 |
| Milk protein conc. | 13.20 ±1.22 | 81.62 ±3.56 | 1.08 ±0.12 | 0.35 ±0.06 | 0.00 ±0.00 | 3.75 ±0.26 |
| Whey protein isolate | 5.80 ±0.86 | 89.26 ±2.48 | 1.90 ±0.32 | 0.32 ±0.01 | 0.00 ±0.00 | 2.72 ±0.68 |
| Sesame | 13.32 ±1.68 | 20.88 ±1.82 | 40.46 ±2.25 | 5.06 ±0.68 | 3.64 ±0.90 | 16.64 ±0.98 |
| Buffalo's ghee | 0.00 ±0.00 | 0.00 ±0.00 | 100 ±0.00 | 0.00 ±0.00 | 0.00 ±0.00 | 0.00 ±0.00 |
| Almond | 4.68 ±0.38 | 15.36 ±1.32 | 65.52 ±3.46 | 3.12 ±0.64 | 6.74 ±0.48 | 4.58 ±.64 |
| Oat | 11.48 ±0.98 | 13.66 ±0.88 | 6.52 ±0.56 | 2.50 ±0.22 | 10.22 ±0.66 | 55.40 ±2.14 |
| Wheat bran | 12.78 ±1.16 | 12.64 ±.84 | 7.22 ±0.36 | 4.42 ±0.20 | 52.56 ±2.53 | 10.38 ±0.98 |
| Peanut butter | 4.08 ±0.66 | 22.82 ±1.34 | 50.20 ±1.48 | 2.36 ±0.18 | 5.12 ±0.32 | 15.42 ±0.88 |
| Coconut powder | 5.40 ±0.42 | 5.82 ±0.76 | 50.66 ±1.88 | 1.06 ±0.08 | 1.54 ±0.12 | 35.52 ±3.12 |
| Date syrup | 29.56 ±2.35 | 1.08 ±0.24 | 0.00 ±0.00 | 2.55 ±0.76 | 3.15 ±0.28 | 63.66 ±1.96 |

[@]: The available carbohydrate was calculated by differences.
